# Shared Human-Chimpanzee Pattern of Perinatal Femoral Shaft Morphology and Its Implications for the Evolution of Hominin Locomotor Adaptations

**DOI:** 10.1371/journal.pone.0041980

**Published:** 2012-07-25

**Authors:** Naoki Morimoto, Christoph P. E. Zollikofer, Marcia S. Ponce de León

**Affiliations:** Anthropological Institute, University of Zurich, Zurich, Switzerland; University of Florence, Italy

## Abstract

**Background:**

Acquisition of bipedality is a hallmark of human evolution. How bipedality evolved from great ape-like locomotor behaviors, however, is still highly debated. This is mainly because it is difficult to infer locomotor function, and even more so locomotor kinematics, from fossil hominin long bones. Structure-function relationships are complex, as long bone morphology reflects phyletic history, developmental programs, and loading history during an individual’s lifetime. Here we discriminate between these factors by investigating the morphology of long bones in fetal and neonate great apes and humans, before the onset of locomotion.

**Methodology/Principal Findings:**

Comparative morphometric analysis of the femoral diaphysis indicates that its morphology reflects phyletic relationships between hominoid taxa to a greater extent than taxon-specific locomotor adaptations. Diaphyseal morphology in humans and chimpanzees exhibits several shared-derived features, despite substantial differences in locomotor adaptations. Orangutan and gorilla morphologies are largely similar, and likely represent the primitive hominoid state.

**Conclusions/Significance:**

These findings are compatible with two possible evolutionary scenarios. Diaphyseal morphology may reflect retained adaptive traits of ancestral taxa, hence human-chimpanzee shared-derived features may be indicative of the locomotor behavior of our last common ancestor. Alternatively, diaphyseal morphology might reflect evolution by genetic drift (neutral evolution) rather than selection, and might thus be more informative about phyletic relationships between taxa than about locomotor adaptations. Both scenarios are consistent with the hypothesis that knuckle-walking in chimpanzees and gorillas resulted from convergent evolution, and that the evolution of human bipedality is unrelated to extant great ape locomotor specializations.

## Introduction

Humans and extant great apes exhibit a pattern of locomotor diversification [1], [2], [3], [4], which stands in contrast with their phyletic relationships. While humans are obligate terrestrial bipeds, our closest living relatives, the chimpanzees, exhibit a wide range of arboreal locomotor behaviors [5], [6], and their peculiar mode of terrestrial quadrupedal locomotion – knuckle-walking – differs substantially from human bipedal locomotion [7], [8]. The more distantly-related gorillas also exhibit various arboreal locomotor behaviors, as well as terrestrial knuckle-walking [9], [10], [11]. Because knuckle-walking occurs in chimpanzees and gorillas, it has been proposed as an ancestral mode of locomotion from which human bipedality evolved [12]. This hypothesis has been challenged on anatomical, developmental and behavioral grounds [13], [14], [15], and the orangutan has been proposed, instead, as a model for the evolution of bipedality from a generalized bipedal/quadrupedal arboreal repertoire of locomotion [14]. In contrast to both hypotheses, the phyletic and functional analysis of the skeleton of *Ardipithecus ramidus* [16], [17], [18], [19], [20] provided evidence that hominin bipedality might have evolved from a locomotor mode no longer present in extant great apes.

During reconstruction of the evolutionary history of hominin bipedalism, fossil evidence from hind limb elements, especially from the femur, has played a central role. The surface topography of the proximal femoral diaphysis of *Ardipithecus ramidus* [16] and *Australopithecus afarensis* [21] has provided evidence for reorganization of the femoropelvic musculature toward bipedal locomotor behaviors [22], [23]. Likewise, the proximal femoral morphology of *Orrorin tugenensis* indicates bipedal locomotor adaptations [24]. Form-function relationships of the femur are complex, however, as femoral morphology results from both long-term processes of selection and adaptation, and short-term processes of bone remodeling during an individual’s lifetime (Wolff’s Law [25] or bone functional adaptation [26], [27]). Femoral morphology thus typically reflects a combination of (a) the impact of an individual’s locomotor history on its musculoskeletal system, (b) taxon-specific adaptation of the musculoskeletal system to specialized locomotor behaviors, and (c) phyletic history not directly related to a taxon’s actual locomotor adaptations (phyletic inertia) [26], [27], [28], [29], [30], [31]. Discrimination between these factors is especially difficult in fossil specimens, for which *in-vivo* patterns of locomotion and species-specific locomotor behavior are unknown, and taxon affiliation is often uncertain.

Here we address these questions by studying femoral morphology in fetuses and neonates of extant great apes and humans. Phyletic relationships and locomotor behaviors of these taxa are well known. Great ape taxa show a remarkable variety of arboreal and terrestrial, quadrupedal and bipedal locomotor behaviors [3], [5], [6], [11], [32], [33], the frequencies of which depend on taxon-specific, environmental and life-history factors [3], [34], [35]. While various modes of terrestrial locomotion are an important component of the locomotor repertoire of chimpanzees and gorillas [11], [33], orangutans are highly restricted to arboreal habitats and are unique among great apes in showing pronograde suspensory behaviors and fist-walking [2], [3].

Studying long bone morphology in fetuses and neonates permits analysis of the effects of the developmental program before the onset of locomotion, that is, before the skeletal morphology is modified by taxon-specific and/or individual mechanical loading regimes, and by environmental factors. Because epiphyses are not yet ossified around the time of birth, we focus on diaphyseal morphology. We ask whether perinatal femoral diaphyseal morphology reflects phyletic relationships independent of an extant taxon’s locomotor adaptation (H0), or whether it reflects adaptation to taxon-specific locomotor behaviors (H1). According to hypothesis H0, humans and chimpanzees should exhibit similar femoral morphologies, to the exclusion of gorillas; according to H1, chimpanzees and gorillas are expected to exhibit largely similar diaphyseal morphologies, while modern human femoral diaphyses should be clearly distinct.

Long bone morphology is brought about by growth in longitudinal and radial directions. During this process, bone is deposited at diaphyseal growth plates and subperiosteal surfaces, respectively, and resorbed at endosteal surfaces [36], [37], [38], [39], [40], [41]. Young et al. [42], [43] have shown that hominoid long bone longitudinal relative to radial growth is more variable than in other primate taxa, and reflects taxon-specific locomotor adaptations. In hominoids, taxon-specific limb proportions are almost fully established at birth [44], indicating distinct taxon-specific longitudinal growth characteristics already before birth. Longitudinal diaphyseal growth characteristics and morphology thus provide support for hypothesis H1.

Here we complement this study by investigating radial diaphyseal morphology. Variability in radial growth results in variability in external (subperiosteal) surface morphology and cortical bone thickness. These features are correlated with musculoskeletal topography [21], [45] and cross-sectional biomechanical properties [46], [47], [48], respectively. Specifically, we ask whether prenatal subperiosteal morphology of the hominoid femoral diaphysis reflects phyletic history (H0) or taxon-specific locomotor adaptations (H1). In the first case (H0), human and chimpanzee morphologies should exhibit several shared-derived features compared to gorilla and orangutan morphologies. In the second case (H1), the fetal/neonate diaphyseal surface morphology of humans should be distinct from that of all great ape taxa, while chimpanzees and gorillas should be more similar to each other than to orangutans.

**Figure 1:**
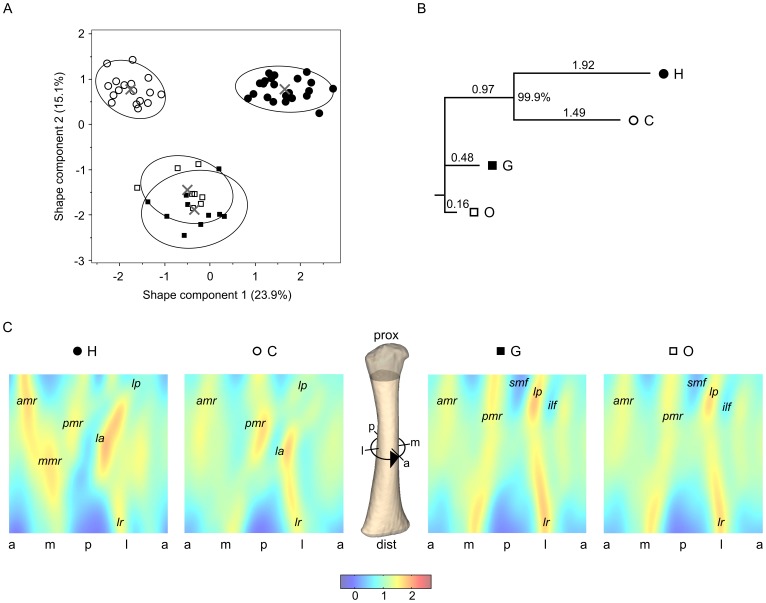
Femoral diaphyseal shape variation in hominoids. A, variation along shape components 1 and 2 of morphospace (humans: filled circles, chimpanzees: open circles, gorillas: filled squares, orangutans: open squares; crosses/ellipses indicate taxon-specific means/90%-density ellipses). B, neighbor-joining tree based on between-taxon distances (see Table 1); numbers above branches indicate branch lengths; number at the branch node indicates bootstrap support (999 of 1000 replications); H: humans, C: chimpanzees, G: gorillas, O: orangutans. C, morphometric maps [false-color images of external surface curvature (relative units)] visualizing taxon-specific mean morphologies (a-m-p-l: anterior-medial-posterior-lateral); *la*: linea aspera, *lp*: lateral pilaster, *ilf*: inferolateral fossa, *smf*: superomedial fossa, *lr*: lateral ridge, *amr*: anteromedial ridge, *pmr*: posteromedial ridge, *mmr*: midshaft medial ridge.

Three-dimensional data of femoral diaphyses were acquired with computed tomography (CT) from a sample of late fetal to neonate humans, chimpanzees, gorillas, and orangutans (see Materials and Methods). Data were analyzed with methods of morphometric mapping (MM), which are well suited to quantify the morphology of relatively featureless cylindroid structures such as long bone diaphyses [29]. In contrast to standard geometric-morphometric techniques, MM does not require pre-defined morphological features such as anatomical landmarks. Rather, morphological features characterizing the sample as a whole, or subsamples, are identified by means of the MM analysis. Here, the shape of the external diaphyseal surface is quantified by its transverse curvature ( = curvature around the shaft), which closely reflects the topography of muscular attachment sites [29], [45] (during the fetal period the internal (endosteal) surface is not yet fully ossified and hence cannot be quantified reliably [37], [49]). Hereafter we use *diaphyseal surface morphology* to denote the resulting MMs (see Materials and Methods, Fig. S1). MMs of all specimens of the sample were aligned so as to minimize differences in rotation around the diaphyseal longitudinal axis. The aligned MMs were then submitted to 2D Fourier Analysis. Principal Components Analysis (PCA) was used to reduce the high dimensionality of the data in Fourier space. This procedure permits to characterize principal patterns of shape variation in the sample (Fig. 1A), to quantify phenetic similarity between taxa [50] (Fig. 1B), and to visualize commonalities and differences between taxon-specific diaphyseal morphologies (Fig. 1C).

## Results and Discussion

MM-based analysis shows that taxon-specific femoral diaphyseal surface morphologies are already present before birth (Fig. 1). Graphing the first two shape components (SC1 and SC2), which account for 23.9% and 15.1% of the total shape variation in the sample indicates that diaphyseal surface morphologies of gorillas (G) and orangutans (O) are more similar to each other than to any other taxon, while diaphyseal morphologies of chimpanzees (C) and humans (H) are approximately equally distant from GO morphologies (Table 1). Differences between taxa along SC1 partly reflect differences in neonatal body mass (Fig. S2A), while differences along shape component 2 are independent of body mass (Fig. S2B). Furthermore, taxon-specific differences in diaphyseal shape are not due to differences in diaphyseal length and cross-sectional area (Fig. S3). Also, sex-specific shape differences could not be found at this early stage of development.

**Table 1:** Morphometric distances between taxon-specific mean shapes.

	H (*Homo*)	C (*Pan*)	G (*Gorilla*)
C (*Pan*)	3.41*	−	−
G (*Gorilla*)	3.32*	3.00*	−
O (*Pongo*)	3.10*	2.57*	0.64 (*p* = 0.41)

* *p*<0.001.

Using orangutans as an outgroup, a phyletic tree evaluated from the data of Fig. 1A clearly groups humans with chimpanzees (HC), versus gorillas (Fig. 1B). Tree topology is well supported by bootstrapping (999 replications of the given tree out of 1000 resamplings). This phene-based tree is consistent with molecular trees of human and great ape phyletic divergence [51], [52], [53], supporting hypothesis H0 that femoral diaphyseal surface morphology in the fetal/neonatal period reflects hominoid phylogeny.

Taxon-specific perinatal femoral diaphyseal surface morphologies are visualized in Fig. 1C. The proximal femoral diaphysis of G and O is characterized by the presence of a prominent lateral spiral pilaster (*lp*) [16], [21], which is delimited by fossae on its inferolateral and superomedial sides (*ilf* and *smf*) [45]. Also, GO femora are characterized by a marked lateral ridge (*lr*) on the distal diaphysis. H and C femoral diaphyses also exhibit a *lp*, but it is only weakly expressed compared to GO. Most notably, the HC femur is characterized by the presence of a linea aspera (*la*) along the posterolateral diaphysis. This feature has a similar position and orientation in humans and chimpanzees, and is not present on GO femora (Fig. 1C).

Which evolutionary processes gave rise to this pattern of morphological similarity and dissimilarity between taxa? Before this question can be addressed, the potential influence of environmental factors and associated loading regimes on fetal long bone development has to be considered. In the uterus, the effects of gravitation are neutralized by buoyancy, but the fetal skeleton experiences loads through spontaneous fetal limb movements, as well as reactive and inertial forces elicited by maternal movements. Clinical evidence shows that spontaneous fetal limb movements are important for normal limb development [54]. These movements are mediated by central pattern generators [55], i.e., genetically programmed neural networks. Fetal movements thus reflect the developmental state of the neuromotor system rather than environmental factors [56]. Also, our results make it unlikely that taxon-specific maternal locomotor/postural behaviors influence fetal long bone morphology. For example, chimpanzee and gorilla neonatal femora have a clearly distinct morphology (Fig. 1) despite largely similar neonatal body size (Table S1) [57], [58], [59] and maternal locomotor behaviors, while gorilla and orangutan neonates have similar femoral diaphyseal morphology, despite significant differences in maternal locomotor behaviors. Overall, it appears unlikely that differences in intrauterine loading regimes contribute substantially to taxon-specific differences in femoral diaphyseal morphology.

**Figure 2:**
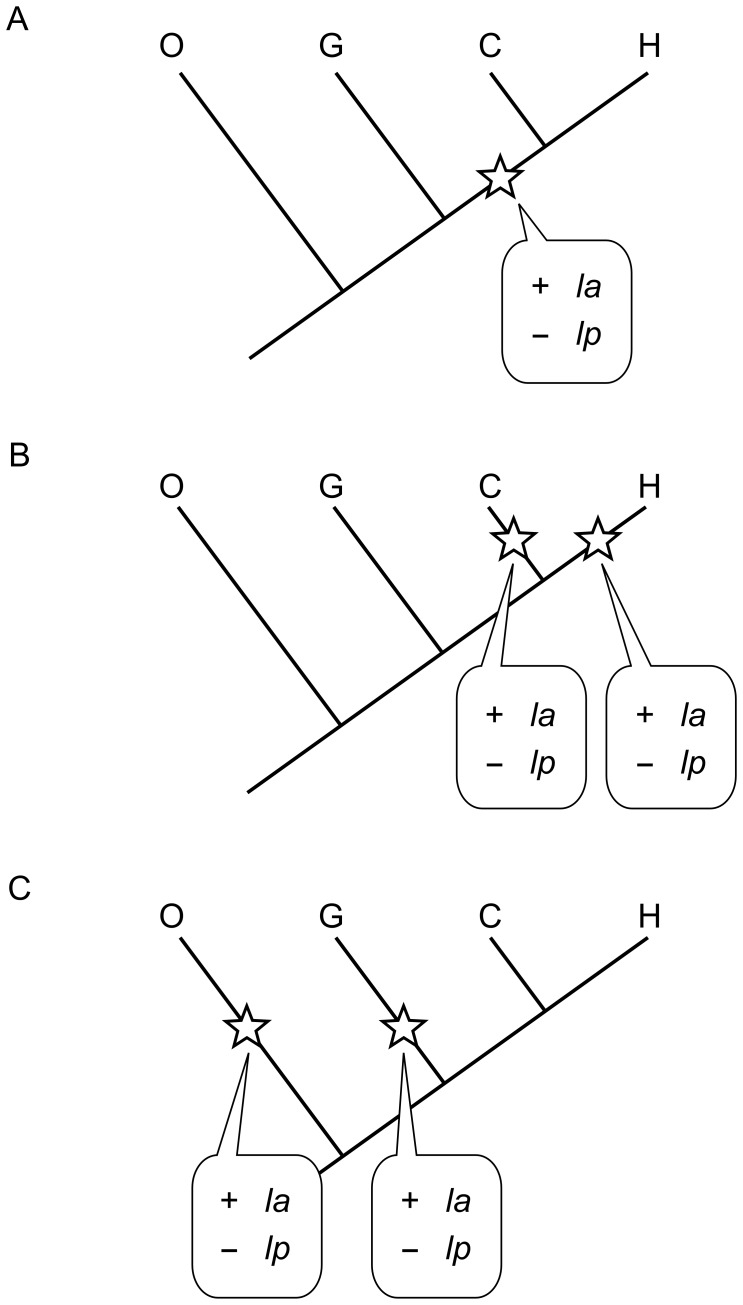
Hypothetical scenarios of femoral diaphyseal shape evolution. Scenario A: shared-derived formation of linea aspera and reduction of lateral pilaster in humans and chimpanzees. Scenario B: parallel evolution of *la* and reduction of *lp*. Scenario C: convergent evolution of similar orangutan/gorilla features (see Fig. 1C for feature codes).

The following evolutionary scenarios bringing about the observed differences between taxa may thus be considered (Fig. 2): (a) H and C similarities in femoral diaphyseal morphology represent shared-derived features, which go back to the last common ancestor (HC-LCA) (Fig. 2A), (b) H and C morphologies evolved independently from an African great ape ancestor (Fig. 2B), and (c) G and O morphologies represent derived states, while the HC-LCA represents the primitive state (Fig. 2C).

Scenarios (b) and (c) imply that similar morphologies result from parallel and convergent evolution, respectively. This is unlikely, given the substantial differences between H and C with respect to locomotor behaviors and associated selective pressures (obligate bipedalism versus predominant quadrupedalism), and between G and O (mostly terrestrial versus predominantly arboreal locomotion).

Scenario (a) is more parsimonious. Adopting this scenario as the most likely one, we may thus infer that, in HC, prenatal femoral diaphyseal ontogeny follows a derived mode, while GO represent the primitive mode. It has been suggested that chimpanzee and gorilla femoral diaphyseal morphologies reflect a shared femoropelvic musculoskeletal organization [16], [21]. In contrast, our results indicate that chimpanzee and gorilla femoral morphologies are distinct already during early development. Together with evidence from musculoskeletal anatomy of ref. [45], this adds to the growing evidence that HC phenetic similarities reflect their close phylogenetic relationship [13], [45], [50], [60]. This is consistent with the hypothesis that knuckle-walking and associated skeletal adaptations of chimpanzees and gorillas evolved independently [15].

It remains to be clarified to which extent the inferred derived HC-LCA diaphyseal surface morphology resulted from neutral evolution (i.e., evolution by drift [61], [62]), and/or from adaptation to taxon-specific locomotor behaviors, respectively. Since close links exist between femoral diaphyseal surface morphology and muscle topography [45], we hypothesize that the HC-LCA underwent an adaptive shift in femoropelvic musculoskeletal organization. Inferences on possible HC-LCA locomotor specialization must remain speculative. If we assume that the posteriorly-located *la* of H and C neonate femora (Fig. 1C) represents a shared-derived feature, its inferred presence in the HC-LCA might indicate a modified function of the muscles inserting along this structure (e.g. the *gluteus maximus*) during hind limb-mediated body propulsion [45].

While our data imply that H and C exhibit shared-derived femoral diaphyseal features relative to G, they also show that morphologies of both H and C diverged from the HC-LCA morphology, probably to a greater extent in H than in C (Figs. 1A,B). This is in concordance with fossil evidence from *Ardipithecus* indicating taxon-specific evolution of femoral morphology not only in hominins but also in panins since their split from the HC-LCA [16], [17], [19], [20].

Human and chimpanzee femoral diaphyseal features unique to each taxon (Figs. 1A,C) most likely reflect taxon-specific locomotor adaptations. For example, humans differ from chimpanzees in exhibiting a prominent anteromedial ridge (*amr*) and a ridge along the medial diaphysis (*mmr*; Fig. 1C) while chimpanzees show a more prominent posteromedial ridge (*pmr*). These morphological differences might reflect differences in the relative size and attachment areas of locomotor muscles around the femur (e.g. large vastus muscles relative to adductor/hamstring muscles in humans compared to chimpanzees [21]). In addition to phyletic divergence, diaphyseal morphologies of H and C also diverge during postnatal development, with the effect that the morphology of the proximal femoral diaphysis of C becomes more similar to G, e.g. regarding the expression of the lateral spiral pilaster (*lsp*) [21], [29]. It remains to be elucidated in greater detail to which extent each of the diaphyseal features identified in Fig. 1C reflects taxon-specific locomotor function, and to which extent they reflect homology versus homoplasy.

Our data provide evidence that the surface morphology of the perinatal hominoid femoral diaphysis reflects phylogenetic affinities (hypothesis H0) to a greater extent than locomotor adaptation (hypothesis H1). The underlying processes of prenatal radial diaphyseal ontogeny appear to be evolutionarily more conservative than those of longitudinal ontogeny. The latter have been shown to reflect taxon-specific locomotor adaptations in terms of limb segment lengths and proportions [42], [43], [44]. While the elongation of the hind limb – which is a key feature of human bipedality [43] – could have been effected by a relatively minor modification of the developmental program [41], radial ontogeny and associated femoral diaphyseal surface morphology seem to be constrained by muscular topography, which has been reported to reflect phyletic relationships in the hominoids [13], [45], [60].

These hypotheses require further testing, especially through detailed comparisons of H, C and G locomotor musculoskeletal development and topography, biomechanics, kinematics, and kinetics. Overall, our results suggest a two-stage approach to investigate the origins of human bipedal locomotion with actualistic data: first identify and analyze the shared-derived features of humans and chimpanzees compared to gorillas, then identify and analyze the uniquely derived features of humans and chimpanzees relative to the inferred HC-LCA, respectively.

## Materials and Methods

### Sample Structure

The sample consists of femora of *Homo sapiens* (*N* = 22; femoral diaphyseal length: 41.6–63.4 mm), *Pan troglodytes* (*N* = 17; 32.2–55.1 mm), *Gorilla gorilla* (*N* = 10; 20.0–59.9 mm) and *Pongo pygmaeus* (*N* = 8; 30.8–46.8 mm) from late fetal stages (3 months pre-term) to neonate stages (before the eruption of the first deciduous molar; <2 months). Since femoral shape does not exhibit significant sex-specific differences at this early stage of development, we used taxon-specific pooled-sex samples. All specimens are from the Collections of the Anthropological Institute and Museum of the University of Zurich.

### Volumetric Data Acquisition

Femora of wet (formalin-preserved, frozen or fresh cadaver) specimens were scanned using a Siemens 64-detector-array CT device (beam collimation 1.0 mm; standard/bone kernels [B30/B60]; serial cross-sections reconstructed at 0.2 mm intervals). Small specimens were scanned using a micro-CT scanner (µCT80, Scanco Medical, Switzerland; volume data reconstructed at an isotropic voxel resolution of 75 µm). Cross sections orthogonal to the principal axis of the femoral shaft were obtained by resampling the original volumetric data using the software Amira 4.1 (Mercury Systems).

### Morphometric Data Acquisition

In immature specimens, unfused epiphyses are often missing, or their position relative to the diaphysis cannot be reconstructed reliably. We thus focus here on diaphyseal morphology. The femoral diaphysis was extracted from the CT volume data using epiphyseal lines as proximal and distal delimiters. Femoral diaphyseal length was measured as the distance between proximal and distal epiphyseal lines. Subperiosteal (external) outlines of each cross section were parameterized with elliptical Fourier analysis (EFA) [63]. EFA was used to reduce noise, and to define parametric outline functions. The curvature of the external diaphyseal surface (*k_ext_*) was calculated analytically using the parametric functions of EFA. Resulting positive/negative values of the curvature *k_ext_* denote convex/concave regions, respectively (see ref. [29] for details).

### Morphometric Analysis

For each specimen, measurements of *k_ext_* were sampled around each cross-sectional outline, and along the entire diaphyseal shaft. These data were normalized to their respective median values, and mapped onto a cylindrical coordinate system (*ρ*, *θ*, *z*), where *ρ* = 1/(2π) = constant denotes the radius of the cylinder. Specimens were prealigned manually such that angle *θ* denotes the anatomical direction (*θ* = 0°→360°: anterior → medial → posterior → lateral → anterior), and *z* denotes the normalized position along the diaphysis (*z* = 0 → 1: distal → proximal) [64], [65]. Since *ρ* = constant, data can be visualized as two-dimensional morphometric maps **M**(*θ*, *z*), and distributions *k_ext_*(*θ*, *z*) (Fig. S1) can be represented as *K*×*L* matrices, where *K* and *L* denote the number of elements along *z* and *θ*, respectively (*K* = *L* = 300).

For the comparative analysis of the morphometric maps **M** *_i_* of all specimens *i* = 1…*N*, differences between specimens in orientation around the diaphyseal long axis (*θ*) had to be minimized. This procedure is analogous to the Procrustes superposition used in anatomical landmark-based geometric morphometric analyses. However, because the morphometric maps of the femoral diaphysis do not contain predefined anatomical features, the alignment was performed in Fourier space. To this end, 2D-Fourier transforms *F*(**M** *_i_*) of all **M** *_i_* were calculated (**M** has a natural periodicity in *θ*), yielding *K*×*L* sets of Fourier coefficients, which define a specimen’s diaphyseal shape as a point in multidimensional Fourier space. Specimens were aligned to each other by minimizing inter-specimen distances in Fourier space through rotation around *θ* (diaphyseal axis).

To reduce the high dimensionality of the data in Fourier space, and to identify principal patterns of shape variability in the sample, Fourier coefficient sets were submitted to Principal Components Analysis (PCA). To facilitate visual inspection and anatomical interpretation of the results of PCA, real-space morphometric maps were reconstructed by transforming a given point P* in PC space into its corresponding set of Fourier coefficients *F*(M*), and applying an inverse Fourier transform to obtain a morphometric map M*. This method was used to produce the MMs of Fig. 1C. Morphometric maps were false-color coded. All calculations were performed with MATLAB7.7 (MathWorks) (see ref. [29] for details).

### Similarity Analysis

Dissimilarity matrices **D** were evaluated to represent all between-taxon distances *D* (quantified as Euclidean distances between taxon mean points; see Table 1) in shape space. Phenetic trees were evaluated for **D** with PHYLIP 3.69 [66], using the neighbor-joining method.

## Supporting Information

Figure S1 Principle of morphometric mapping. A, 3D representation of the right femur. B, principle of cylindrical projection (anterior [0°] → medial [90°] → posterior [180°] → lateral [270°] → anterior [0°]).
(TIF)

Figure S2 Correlation between taxon-specific means of shape component scores and means of neonatal body mass (data summarized in Table S1; humans: filled circles, chimpanzees: open circles, gorillas: filled squares, orangutans: open squares). SC1 is weakly correlated with neonatal body mass (*p* = 0.06, *R* ^2^ = 0.88) (A). SC2, which distinguishes between human-chimpanzee and gorilla-orangutan, is not correlated with neonatal body mass (B).
(TIF)

Figure S3 Correlation between femoral diaphyseal shape component scores (SC1, SC2) and femoral size (humans: filled circles, chimpanzees: open circles, gorillas: filled squares, orangutans: open squares). Shape component scores are plotted against femoral diaphyseal length (A), and median femoral diaphyseal cross-sectional area (B). Each cross-sectional area was calculated as the total area of bone marrow-filled cross-section. Overall, taxon-specific differences in femoral diaphyseal length are not correlated with femoral diaphyseal morphology. Humans exhibit a weak correlation of SC1 with femoral diaphyseal length (*p*<0.05, *R* ^2^ = 0.20); chimpanzees exhibit a weak correlation of SC1 with femoral diaphyseal cross-sectional area (*p*<0.05, *R* ^2^ = 0.28).
(TIF)

Table S1 Neonatal body mass of hominoids.
(DOCX)
