# Supplementary figures and images for: Shared Human-Chimpanzee Pattern of Perinatal Femoral Shaft Morphology and Its Implications for the Evolution of Hominin Locomotor Adaptations
Source: PLoS One. 2012 Jul 25;7(7):e41980. doi: 10.1371/journal.pone.0041980 (PMC3405051; doi:10.1371/journal.pone.0041980)

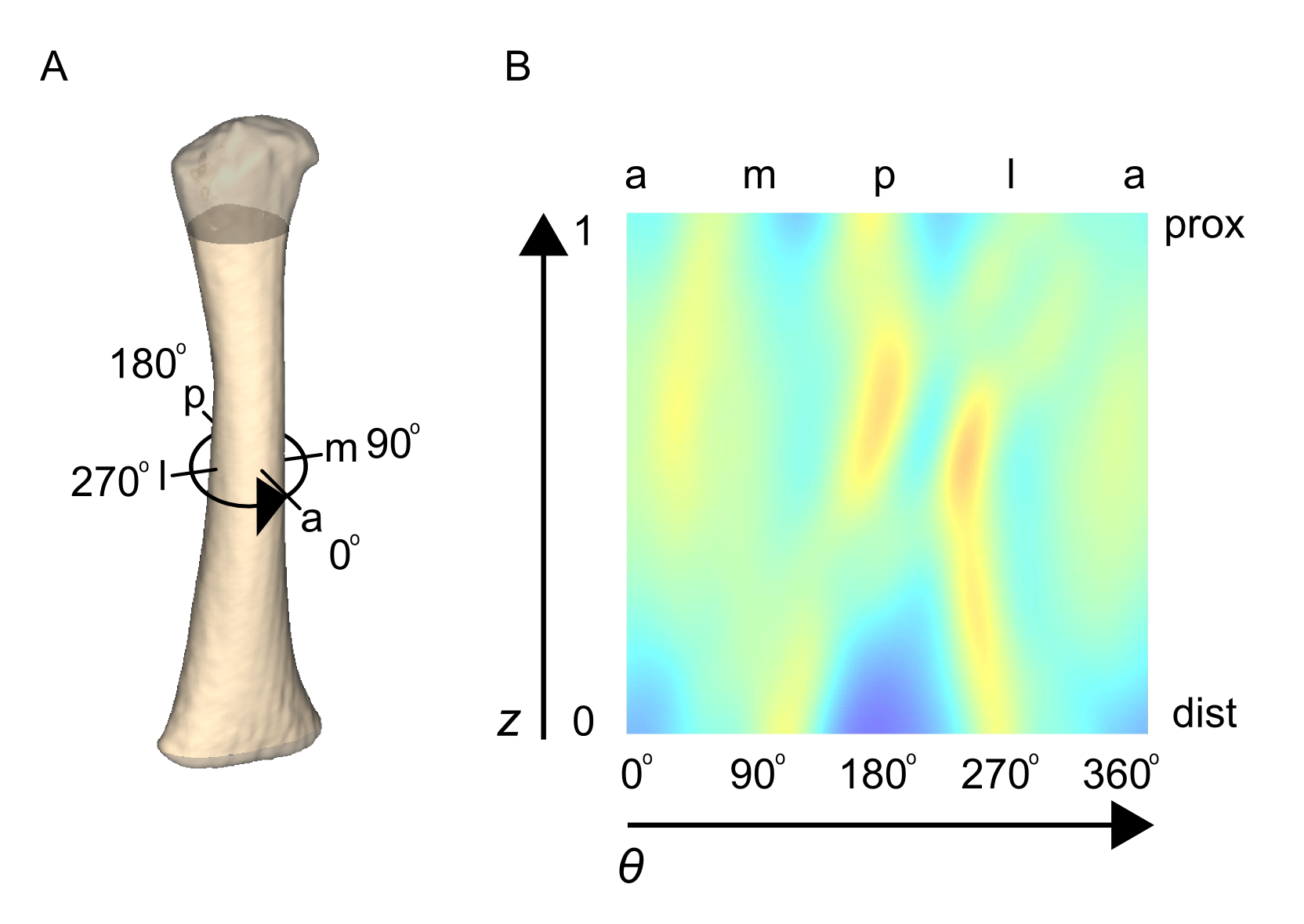

Supplement: Figure S1 — Principle of morphometric mapping. A, 3D representation of the right femur. B, principle of cylindrical projection (anterior [0°] → medial [90°] → posterior [180°] → lateral [270°] → anterior [0°]). (TIF) [file pone.0041980.s001.tif]

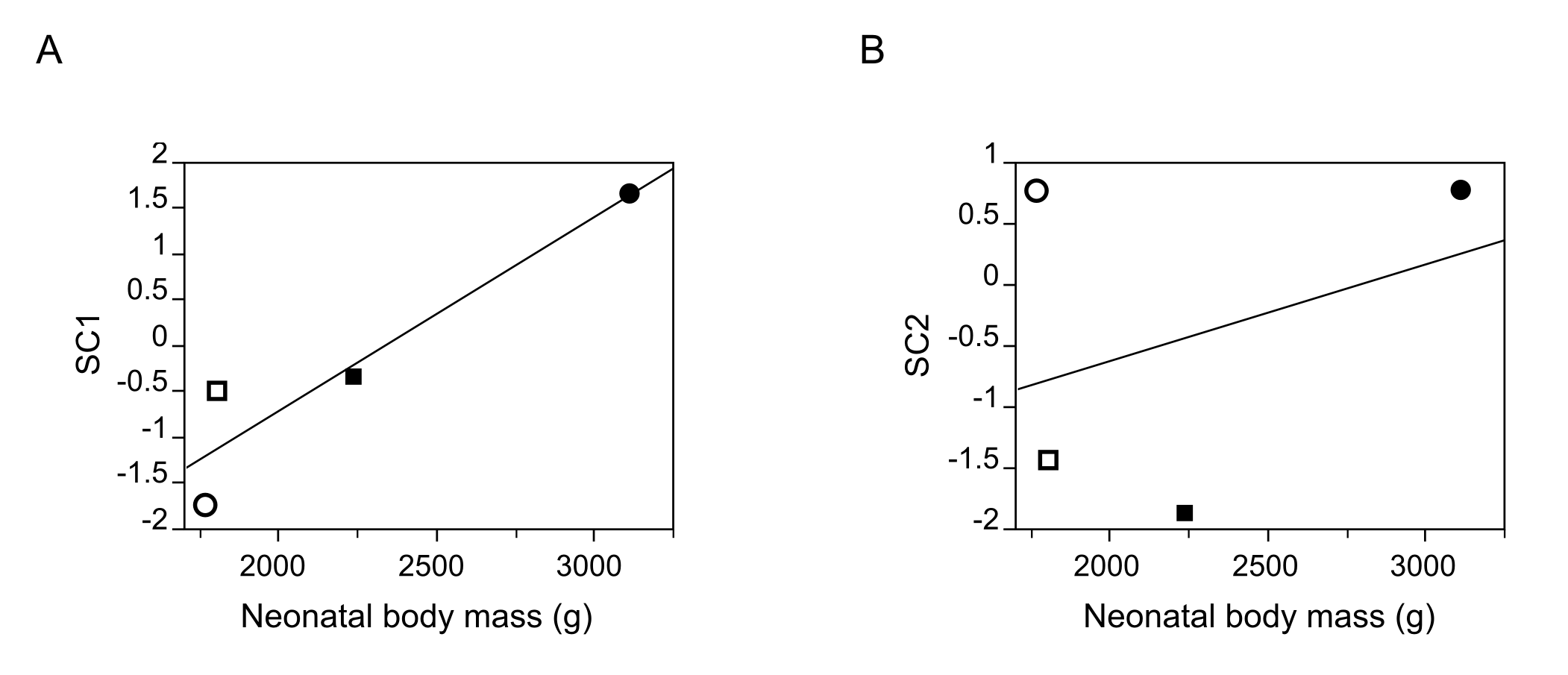

Supplement: Figure S2 — Correlation between taxon-specific means of shape component scores and means of neonatal body mass (data summarized in Table S1; humans: filled circles, chimpanzees: open circles, gorillas: filled squares, orangutans: open squares). SC1 is weakly correlated with neonatal body mass (p = 0.06, R 2 = 0.88) (A). SC2, which distinguishes between human-chimpanzee and gorilla-orangutan, is not correlated with neonatal body mass (B). (TIF) [file pone.0041980.s002.tif]

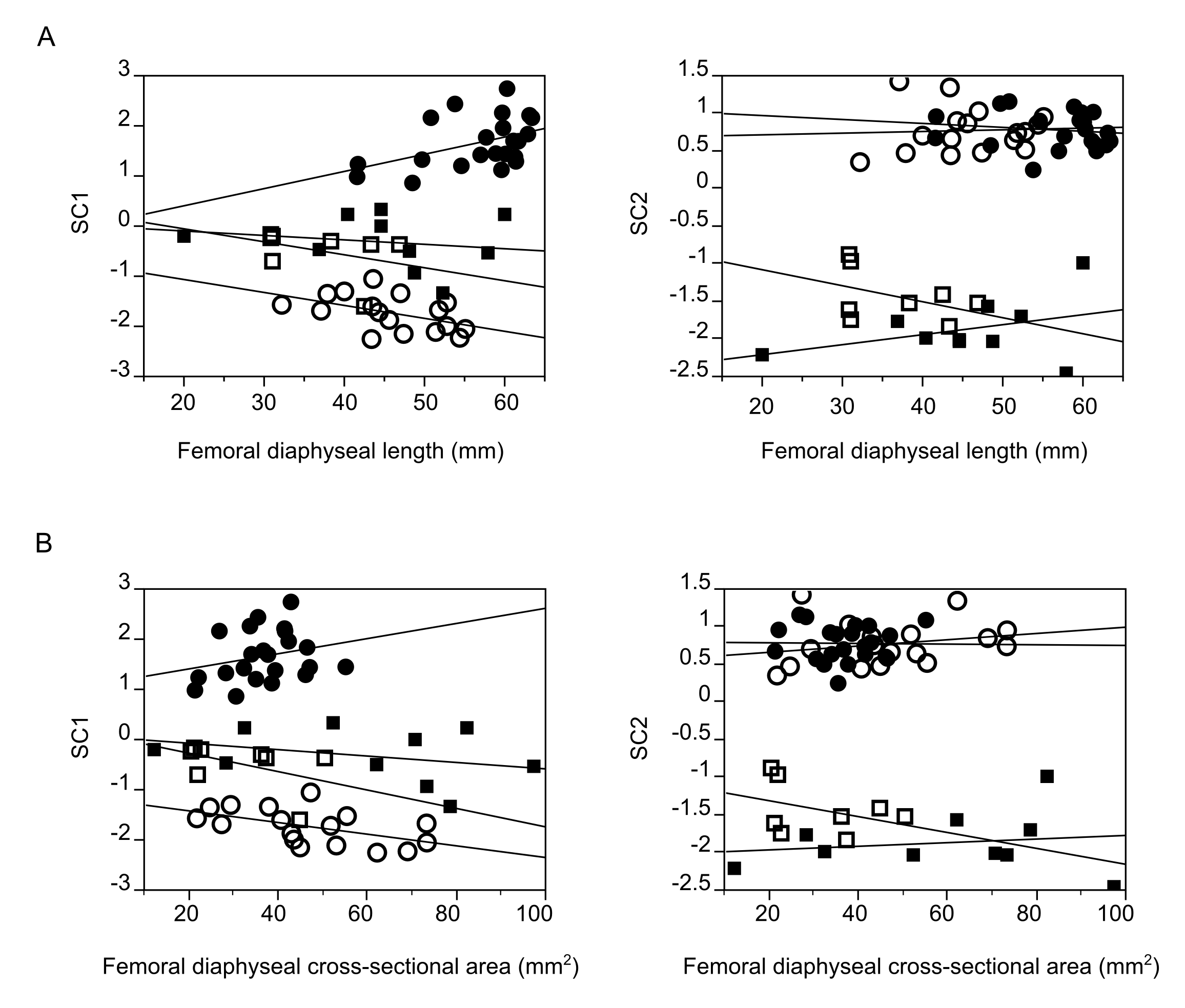

Supplement: Figure S3 — Correlation between femoral diaphyseal shape component scores (SC1, SC2) and femoral size (humans: filled circles, chimpanzees: open circles, gorillas: filled squares, orangutans: open squares). Shape component scores are plotted against femoral diaphyseal length (A), and median femoral diaphyseal cross-sectional area (B). Each cross-sectional area was calculated as the total area of bone marrow-filled cross-section. Overall, taxon-specific differences in femoral diaphyseal length are not correlated with femoral diaphyseal morphology. Humans exhibit a weak correlation of SC1 with femoral diaphyseal length (p<0.05, R 2 = 0.20); chimpanzees exhibit a weak correlation of SC1 with femoral diaphyseal cross-sectional area (p<0.05, R 2 = 0.28). (TIF) [file pone.0041980.s003.tif]
