# Supplementary material for: Shared Human-Chimpanzee Pattern of Perinatal Femoral Shaft Morphology and Its Implications for the Evolution of Hominin Locomotor Adaptations
Source: PLoS One. 2012 Jul 25;7(7):e41980. doi: 10.1371/journal.pone.0041980 (PMC3405051; doi:10.1371/journal.pone.0041980)
Supplement: Table S1 — Neonatal body mass of hominoids. (DOCX) [file pone.0041980.s004.docx]

**Table S1 Neonatal body mass of hominoids**

| **Taxon** | **Body mass (g)** | ***N*** | **Source** |
| --- | --- | --- | --- |
| *Homo sapiens* | 3111.3* | 11317 | DeSilva, 2011 [[1](#_ENREF_1)] |
| *Pan troglodytes* | 1766 | 45 | Leigh and Shea, 1996 [[2](#_ENREF_2)] |
| *Pan troglodytes* | 1766 | 68 | DeSilva, 2011 |
| *Pan troglodytes* mean | 1766* | 113 |  |
| *Gorilla gorilla* | 2327 | 136 | Leigh and Shea, 1996 |
| *Gorilla gorilla* | 2251(m), 1996 (f) | 56, 55 | Smith and Leigh, 1998 [[3](#_ENREF_3)] |
| *Gorilla gorilla* mean | 2236.1* | 247 |  |
| *Pongo pygmaeus* | 1965 (m), 1653 (f) | 27, 28 | Smith and Leigh, 1998 |
| *Pongo pygmaeus* mean | 1806.2* | 55 |  |

*used in Fig. S2

**References**

1. DeSilva JM (2011) A shift toward birthing relatively large infants early in human evolution. Proceedings of the National Academy of Sciences of the United States of America 108: 1022-1027.

2. Leigh SR, Shea BT (1996) Ontogeny of body size variation in African apes. Am J Phys Anthropol 99: 43-65.

3. Smith RJ, Leigh SR (1998) Sexual dimorphism in primate neonatal body mass. Journal of Human Evolution 34: 173-201.
